# Supplementary material for: Probiotic Bacteria from Human Milk Can Alleviate Oral Bovine Casein Sensitization in Juvenile Wistar Rats
Source: Microorganisms. 2023 Apr 14;11(4):1030. doi: 10.3390/microorganisms11041030 (PMC10140966; doi:10.3390/microorganisms11041030)
Supplement: Supplementary file 1 [file microorganisms-11-01030-s001.zip › microorganisms-2286679-supplementary.pdf]

microorganisms

Table S1. Sequences producing significant alignments for SL42 isolate compared to other species in NCBI GenBank.

Description	Scientific Name	Max Score	Total Score	Query Cover	E value	Per. Ident	Acc. Len	Accession
Lacticaseibacillus rhamnosus strain SL42 16S ribosomal RNA gene, partial sequence	Lacticaseibacillus rhamnosus	798	798	100%	0.0	100.00%	432	OQ300076.1
Lacticaseibacillus rhamnosus strain 3030 16S ribosomal RNA gene, partial sequence	Lacticaseibacillus rhamnosus	782	782	99%	0.0	99.53%	1472	MT613447.1
Uncultured Lactobacillus sp. clone s5 16S ribosomal RNA gene, partial sequence	uncultured Lactobacillus sp.	780	780	99%	0.0	99.31%	1481	HM151341.1
Lactobacillus paracasei strain 3099 16S ribosomal RNA gene, partial sequence	Lacticaseibacillus paracasei	778	778	98%	0.0	99.76%	1464	MT613487.1
Lacticaseibacillus rhamnosus strain 3034 16S ribosomal RNA gene, partial sequence	Lacticaseibacillus rhamnosus	778	778	98%	0.0	99.76%	1479	MT613451.1
Lacticaseibacillus rhamnosus strain 2418 16S ribosomal RNA gene, partial sequence	Lacticaseibacillus rhamnosus	778	778	98%	0.0	99.76%	1471	MT604852.1

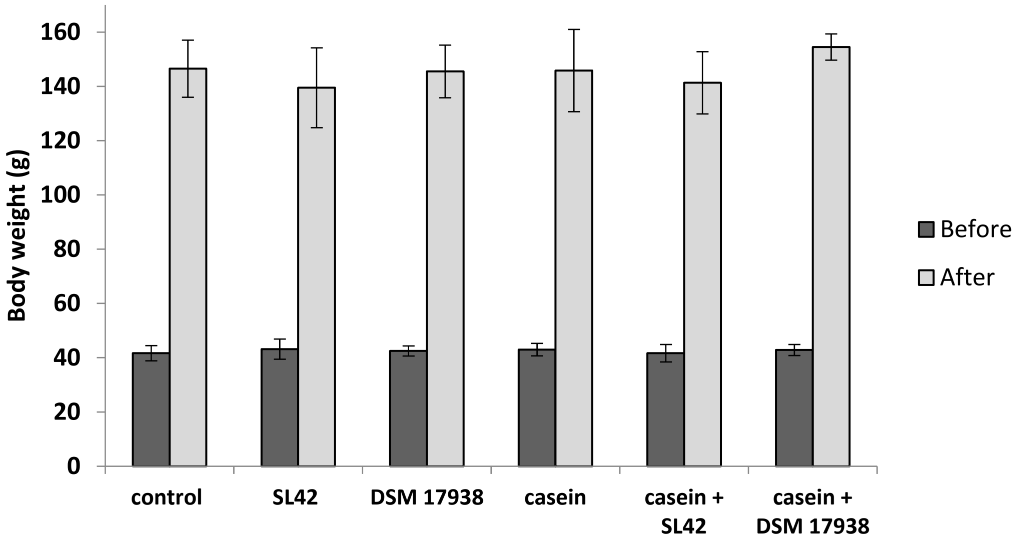

Figure S1. Body weight variation (g) before (day 1st), and after (day 58th) CAS-challenge. The values are shown as the means ± SD (n = 8 rats/group). No significant differences were observed at $P > 0.05$.

Table S2. Body temperature (°C) variation during CAS-challenge. The values are shown as the means ± SD (n = 8 rats/group). No significant differences were observed at *P* > 0.05.

	Control	SL42	DSM 17938	Casein	Casein + SL42	Casein + DSM17938
30 min	37 ±0.21	36 ±0.43	37 ±0.1	36.5 ±0.43	36 ±0.43	36 ±0.21
60 min	37 ±0.3	37 ±0.21	37 ±0.2	36 ±0.21	36 ±0.43	36 ±0.21
120 min	37 ±0.2	36.5 ±0.21	37 ±0.23	37 ±0.43	37 ±0.5	37 ±0.43

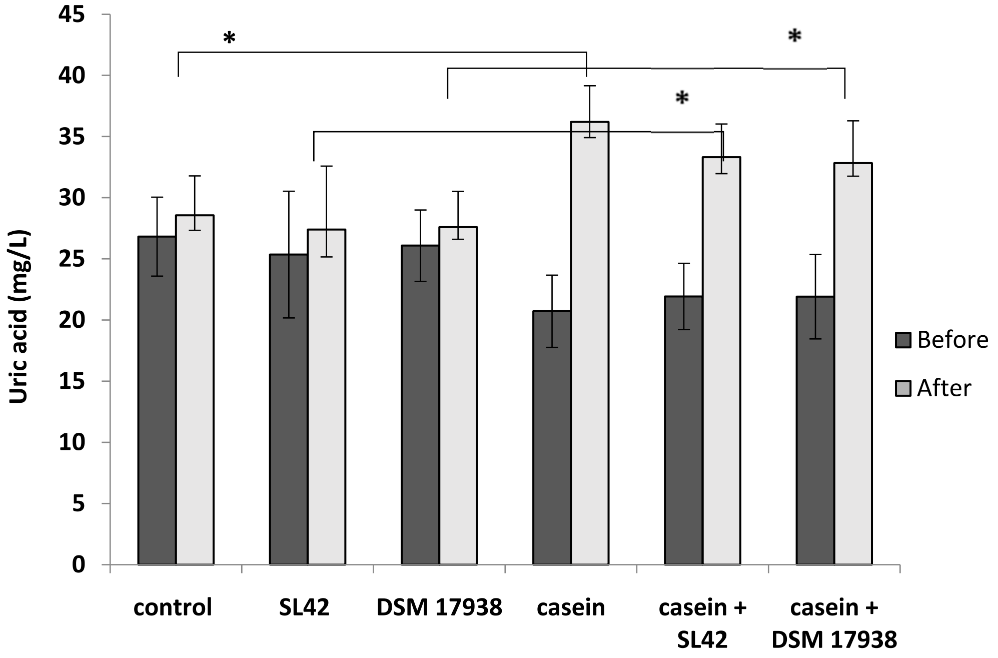

Figure S2. Uric acid levels in sera of rats before (day 1st), and after (day 58th) CAS-challenge. The values are shown as the means ± SD (n = 8 rats/group). * Significant differences were observed at *P* < 0.05.
